# Advantages and Versatility of Fluorescence-Based Methodology to Characterize the Functionality of LDLR and Class Mutation Assignment

**DOI:** 10.1371/journal.pone.0112677

**Published:** 2014-11-11

**Authors:** Aitor Etxebarria, Asier Benito-Vicente, Ana C. Alves, Helena Ostolaza, Mafalda Bourbon, Cesar Martin

**Affiliations:** 1 Unidad de Biofísica (CSIC, UPV/EHU) and Departamento de Bioquímica y Biología Molecular, Universidad del País Vasco, Apdo. 644, 48080 Bilbao, Spain; 2 Grupo de Investigação Cardiovascular, Unidade I&D, Departamento de Promoção da Saúde e Prevenção de Doenças Não Transmissíveis, Instituto Nacional de Saúde Dr. Ricardo Jorge, Lisboa 1649-040, Portugal; 3 Centre for Biodiversity, Functional and Integrative Genomics, Faculty of Sciences, University of Lisboa, 1749-016 Lisboa, Portugal; Centro Cardiologico Monzino, Italy

## Abstract

Familial hypercholesterolemia (FH) is a common autosomal codominant disease with a frequency of 1∶500 individuals in its heterozygous form. The genetic basis of FH is most commonly mutations within the *LDLR* gene. Assessing the pathogenicity of LDLR variants is particularly important to give a patient a definitive diagnosis of FH. Current studies of LDLR activity *ex vivo* are based on the analysis of ^125^I-labeled lipoproteins (reference method) or fluorescent-labelled LDL. The main purpose of this study was to compare the effectiveness of these two methods to assess LDLR functionality in order to validate a functional assay to analyse *LDLR* mutations. LDLR activity of different variants has been studied by flow cytometry using FITC-labelled LDL and compared with studies performed previously with ^125^I-labeled lipoproteins. Flow cytometry results are in full agreement with the data obtained by the ^125^I methodology. Additionally confocal microscopy allowed the assignment of different class mutation to the variants assayed. Use of fluorescence yielded similar results than ^125^I-labeled lipoproteins concerning LDLR activity determination, and also allows class mutation classification. The use of FITC-labelled LDL is easier in handling and disposal, cheaper than radioactivity and can be routinely performed by any group doing LDLR functional validations.

## Introduction

Familial hypercholesterolemia (FH; MIM #143890) was the first genetic disease of lipid metabolism to be characterized and is mechanistically linked to the pathogenesis of coronary heart disease (CHD) [1]. FH is an autosomal codominant disorder characterized by increased plasma LDL cholesterol, tendon xanthomas, deposits of cholesterol in peripheral tissues and accelerated atherosclerosis, leading to premature coronary heart disease (CHD) [1]. FH has a heterozygous frequency of 1∶500 and a homozygous frequency of 1∶1,000,000, with a prevalence of 1∶200,000 for compound heterozygous [2]. FH is mainly due to mutations in the LDL receptor (*LDLR*; MIM #606945) gene, which is responsible for the uptake of LDL particles into cells [1]. To date, more than 1,300 different mutations in the *LDLR* gene have been identified worldwide [3] but not all of them have been proved to affect receptor activity. Mutations have been assigned to different classes depending on their effect: class 1 mutations result in no detectable LDL receptor protein [4]; class 2 mutations cause either complete (class 2a) or partial (class 2b) block of transport of the LDL receptor from the ER [5]; class 3 mutations result in defective LDL binding [6]; class 4 mutations cause defective clustering in clathrin coated pits [7], [8] and class 5 mutations result in recycling defective receptors [9]. In order to provide a definite and complete diagnosis of FH, *LDLR* mutations should be assessed both for activity and class type mutation.

In a recently reported study, *in silico* analysis of 443 described missense variants predicted a loss of LDLR activity in 89% of the described variants using at least one *in silico* method [10]. However, it is well recognized that *in silico* software has several limitations and has to be used with caution [11]. Functional validation of these LDLR variants must be assessed in order to identify which mutations lead to a functional loss of receptor activity. Once characterized, unequivocal genetic diagnosis of FH is possible, allowing the implementation of adequate treatment (either early initiation or intensification) to reduce the increased cardiovascular risk characteristic of these patients. The early identification and treatment of FH patients is especially important for young patients in whom the physical stigmata are not yet developed and so CHD can be prevented. Current methodologies, which determine LDLR functionality ex* vivo*, are based on the use of radioactivity or flow cytometry. LDL uptake and degradation of ^125^I-labeled LDL is commonly determined in radioactivity-based methods, and very similarly, binding and uptake of LDL are determined by flow cytometry when using fluorescent-labelled LDL [6], [12]–[17]. Both methods are used indistinctly, probably depending on the research laboratory facilities or because there is a traditional used methodology in the laboratory.

To compare the suitability of both techniques in the determination of LDLR activity with regard to sensitivity, reproducibility and practical considerations, we have conducted an inter-laboratory comparative study. Data obtained by ^125^I assays in one of the laboratories [11] have been compared with data obtained by FITC-labelled LDL in another laboratory. We have also analysed whether the use of FITC-labelled LDL and flow cytometry is a reliable assay for the functional characterization of LDLR variants and whether the advantages of the fluorescence assay can be extended to the assignment of LDLR class type mutation. In both type of experiments CHO-*ldlA7* cells transfected with the plasmids encoding wild-type (wt) or *LDLR* variants p.Val429Leu (c.1285G>C), p.Trp490Arg (c.1468T>C), p.Ser648Pro (c.1942T>C), p.Pro685Ser (c.2053C>T) and p.Val859Met (c.2575G>A) have been used. Confocal laser microscopy was used to determine the cellular localization, class type mutation and biological activity of the mutant LDLR.

## Materials and Methods

### Variant nomenclature

For sequence analysis the reference sequence used was NM_000527.4 and cDNA numbering was considered following the Human Genome Variation Society nomenclature, with nucleotide c.1 being A of the ATG initiation codon p.1 [18], [19].

### *In silico* analysis

The predicted effects of *LDLR* alterations were assessed using the following open access software: PolyPhen-2 [20], Sorting Tolerant From Intolerant (SIFT) [21], Consensus Deleteriousness score of missense SNVs (Condel) [22], Mutation taster [23], Grantham Score [24] and PhyloP [25]. The PolyPhen-2 programs give predictions of “Probably Damaging”, “Possibly Damaging” and “Benign”. The programs SIFT and CONDEL predict whether an amino acid substitution would be “Deleterious” or “Neutral”. The program Mutation Taster gives a prediction of “Disease Causing” or “Polymorphism”. The program Grantham Score predicts, in a numerical form, whether an amino acid substitution is: conservative (0–50), moderately conservative (51–100), moderately radical (101–150), or radical (≥151). The program PhyloP (phylogenetic p-value) gives a prediction in a numerical form between −14.1 and 6.4 (the higher the value the most conserved the nucleotide is between species).

### Site-directed mutagenesis

Individual point mutations leading to p.Val429Leu, p.Trp490Arg, p.Ser648Pro, p.Pro685Ser and p.Val859Met LDLR variants were generated as described before [11]. Presence of the desired nucleotide alteration was confirmed by PCR and restriction enzyme digestion of the appropriate fragments and the integrity of the remaining LDLR cDNA sequence of all constructs was verified by direct sequence analysis.

### Expression of LDLR proteins *in vitro*

LDLR-deficient Chinese hamster ovary (CHO) cell line *ldlA7* (CHO-*ldlA7*) (kindly provided by Dr. Monty Krieger, Massachusetts Institute of Technology, Cambridge, MA) was cultured in Ham’s F-12 medium supplemented with 5% FBS, 2 mM L-glutamine, 100 units/mL penicillin, and 100 µg/mL streptomycin. CHO-*ldlA7* cells were plated into 24 well culture plates, and transfected with plasmids carrying the *LDLR* mutations using Lipofectamine LTX and Plus Reagent (Invitrogen) according to the manufacturer’s instructions. Transfected cells were maintained in culture during 48 h to achieve maximal LDLR expression.

### Western blot analysis

Cell lysates were prepared, protein concentration determined, and fractionated by electrophoresis on non-reducing 8.5% SDS-PAGE for semi-quantitative immunoblotting. Membranes were immunostained with rabbit polyclonal anti-LDLR antibody (1∶2000) (Cayman Chemical, Cat. No. 10007665) for 16 h at 4°C and rabbit polyclonal IgG anti-GAPDH antibody (1∶1000) (Santa Cruz Biotechnology, Cat. No. SC-25778) for 1 h at room temperature and counterstained with a horseradish peroxidase-conjugated anti-rabbit antibody (Cell Signalling, Cat. No: 7074s). The signals were developed using SuperSignal West Dura Extended Substrate (Pierce Biotechnology, Rockford, IL, USA). ChemiDoc XRS (Bio-Rad, Hercules, CA, USA) was used to detect the signals, and Quantity One Basic 4.4.0 software (Bio-Rad) was used to quantify band intensities. The concentrations of the antibodies were optimized to achieve low background and a linear dose-dependent increase in signal intensity. The relative band intensities for the mature and precursor forms of LDLR protein expressed for the different constructs was calculated as the ratio between the LDLR 160 kDa or 130 kDa bands to that of GAPDH.

### Real-time Quantitative PCR

CHO-*ldlA7* cells (0.5×10^6^) were seeded in 6-well tissue-culture plates overnight and transfected as described before. 48 h post-transfection, RNA was harvested from cells using TRIzol Reagent (Invitrogen), and cDNA was synthesized from 1 µg of RNA using Affinity Script qRT-PCR cDNA synthesis kit (Stratagene, Agilent Technologies, USA) according to the manufacturer’s instructions. The qRT-PCR was performed in triplicates using the Brilliant-III Ultra Fast SYBR Green QPCR. Primers were purchased from Integrated DNA Technologies (Cat. No. Hs.PT.58.3621384 for LDLR and, the primer sequences used to amplify hamster GAPDH gene were 5′-CATGTTCCAGTATGACTCCACTC-3′ and 5′-GGCCTCACCCCATTTGATGT-3′). Reactions were performed in the Applied Biosystems 7900HT Fast Real-Time PCR System, the real-time PCR program consisted of 40 cycles (95°C for 15 s and 60°C for 1 min) after initial 10 min incubation at 95°C. Reactions were conducted in triplicate, and expression of all transcripts relative to GAPDH was determined.

### Uptake and degradation of ^125^I-LDL

LDL catabolism in CHO-*ldlA7* cells carrying wt or LDLR variants was determined by using ^125^I-labelled LDL as previously described [12], [26]. Each LDLR variant expressing CHO-*ldlA7* cells were seeded on day 1 in 12-well plates (2×10^5^ cells/dish) in triplicates to determine the ability of uptake (binding plus internalization) or degrade ^125^I-labelled LDL. To achieve maximum LDLR expression, on day 2 cells were washed and preincubated for 12 h in growth medium containing 10% (v/v) lipoprotein-deficient serum (LPDS, Chemicon) and sterols (90 µg/mL of cholesterol and 9 µg/mL of 25(OH)-cholesterol). On day 3, cells were washed and incubated for 4 h at 37°C in medium containing 5% LPDS and different concentrations of ^125^I-LDL. LDL uptake, determined at 37**°**C, accounts for both binding and internalization of LDL particles. Saturable uptake and degradation of ^125^I-LDL were determined as the difference in cell-associated radioactivity (uptake) or the acid-soluble non-iodine radioactivity in the medium (degradation) between cells incubated with ^125^I-LDL in the presence or absence of an excess (1 mg/mL) of unlabeled LDL, as described before [16], [27]. Experiments were repeated at least twice with triplicate samples for each cell line. Data is shown as the percentage of maximum obtained for wt LDLR at 4 h.

### Lipoprotein labelling with FITC

LDL was labelled with FITC as previously described [28]. Briefly, LDL (1 mg/mL) in 0.1 M NaHCO_3_ (pH 9.0) were mixed with 10 µl/mL of FITC (2 mg/mL in dimethyl sulfoxide). The mixture was gently mixed by slow rocking at room temperature for 2 h. The unreacted dye was removed by gel filtration on a sephadex G-25 column equilibrated with PBS EDTA-free buffer. All fractions were assayed for protein content with bovine serum albumin as standard (Pierce BCA protein assay, Pierce).

### Quantification of LDLR activity by flow cytometry

Transfected CHO-*ldl*A7 cells were grown in 24 well culture plates. 48 h after transfection, cells were incubated for 4 h, at 37°C or at 4°C with 20 µg/mL FITC-LDL to determine LDLR activity or LDL-LDLR binding, respectively. After incubation with FITC-LDL, CHO-*ldl*A7 cells were washed twice in PBS-1%BSA, fixed on 4% formaldehyde for 10 min and washed again twice with PBS-1%BSA. The quenching of external fluorescence, which distinguishes internalized from surface-adherent FITC-LDL particles, can be accomplished with the use of vital dyes such as Trypan blue [29], which is not able of penetrating intact cell membranes. This procedure allows to remove extracellular fluorescence by quenching and to determine the intensity of the remaining fluorescent particles inside the cells that is not affected by the external quencher. Therefore, to determine the amount of internalized LDL, Trypan blue solution (Sigma-Aldrich, Steinheim, Germany) was added to a final concentration of 0.2% directly to the samples, eliminating the extracellular signal due to the non internalized LDLR-LDL complexes. Fluorescence intensities were measured by flow cytometry, in a Facscalibur Flow cytometer according to the manufacturer instructions as previously described [30]. For each sample, fluorescence of 10,000 events was acquired for data analysis. All measurements have been performed at least in triplicate. Uptake efficiency was corrected using the data of mature protein expression quantified by Western blot and data is shown as the percentage of maximum obtained for wt LDLR.

### Quantification of LDLR expression by flow cytometry

To determine cell surface expression of LDLR by FACS, transfected CHO-*ldl*A7 cells grown during 48 h were incubated with a mouse primary antibody anti-LDLR (1∶100; 5 mg/L; Progen Biotechnik GmbH, Cat. No. 61087) for 1 h, at room temperature, then, washed twice with PBS-1%BSA and incubated with a secondary antibody Alexa Fluor 488-conjugated goat anti-mouse IgG (1∶100; Molecular Probes; Cat. No. A-11001). As negative controls, non transfected CHO-*ldl*A7 cells or transfected with the wt LDLR were stained with the same primary anti-LDLR antibody (Progen Biotechnik GmbH, Cat. No. 61087), which is negative for CHO-*ldl*A7 cells and, with mouse IgG2b, kappa monoclonal [MPC-11]-isotype control (abcam; Cat. No. ab18457) in identical conditions. As shown in Figure S1, isotype control antibody has no specificity for CHO-*ldl*A7 cells transfected or not with LDLR. For each sample, fluorescence of 10,000 events was acquired for data analysis. All measurements have been performed at least in triplicate. LDLR expression efficiency was corrected using the data of mature protein expression quantified by Western blot and data is shown as the percentage of maximum obtained for wt LDLR.

### Confocal Laser Scanning Microscopy (CLSM)

CLSM was used to analyze expression of LDLR and colocalization of LDLR with endoplasmic reticulum (ER). Briefly, cells grown in coverslips were transfected with the *LDLR* containing plasmids and cultured for 48 h, at 37°C in 5% CO_2_. Then, the medium was removed and glass slides washed twice with PBS-1%BSA. For these studies non labelled lipoproteins (20 µg/mL LDL) were added and cells were incubated at 37°C for additional 4 h. Cells were fixed with 4% paraformaldehyde during 10 min and washed three times with PBS-1%BSA and permeabilized with 1% TritonX-100 for 30 min at room temperature. Samples were then washed and blocked in PBS-10% FBS for 1 h and washed in PBS-1%BSA three times. Then, samples were incubated for 16 h at 4°C with the appropriate primary antibodies: chicken pAB anti-calreticulin (abcam; Cat. No. ab18457), and mouse mAB anti-LDLR (Progen Biotechnik GmbH, Cat. No. 61087) for calreticulin and LDLR colocalization; and, Rabbit pAB Anti-Apolipoprotein B (abcam; Cat. No. ab20737) for ApoB-100 and LDLR colocalization; followed by incubation with the appropriate fluorescent secondary antibodies: Alexa Fluor 594 Goat Anti-Chicken IgG (Molecular probes; Cat. No. sc-11042), Alexa Fluor 488 Rabbit Anti-Mouse IgG (Molecular probes; Cat. No. A-11059), Texas Red goat anti-mouse IgG (Molecular probes; Cat. No. T-862) or Alexa Fluor 488 Goat Anti-Rabbit IgG (Molecular probes; Cat. No. A-11008). Coverslips were mounted on a glass slide and samples were visualized using a confocal microscope (Olympus IX 81) with sequential excitation and capture image acquisition with a digital camera (Axiocam NRc5, Zeiss). Images were processed with Fluoview v.50 software.

### Statistical analysis

All measurements were performed at least 3 times, with n = 3 unless otherwise stated, and results are presented as mean ± standard deviation (S.D.). Levels of significance were determined by a two-tailed Student’s t-test, and a confidence level of greater than 95% (p<0.05) was used to establish statistical significance.

## Results

### *In silico* analysis

The results obtained by different software packages are presented in Table 1.

**Table 1:** Results obtained by the different bioinformatics tools for each alteration under study.

				Pathogenicity prediction			
cDNA	Protein	phyloP	Grantham score	SIFT	Polyphen-2	Mutation Taster	CONDEL
c.1285G>C	p.Val429Leu	3.84	Conservative	Tolerated	Benign	Disease Causing	Neutral
c.1468T>C	p.Trp490Arg	4.48	Moderate radical	Deleterious	Probably damaging	Disease Causing	Deleterious
c.1942T>C	p.Ser648Pro	0.93	Moderate conservative	Deleterious	Probably damaging	Polymorphism	Neutral
c.2053C>T	p.Pro685Ser	5.72	Moderate conservative	Deleterious	Probably damaging	Disease Causing	Deleterious
c.2575G>A	p.Val859Met	−0.76	Conservative	Deleterious	Probably damaging	Polymorphism	Deleterious

### Expression of LDLR variants in CHO-*ldl*A7 cells

**Figure 1:**
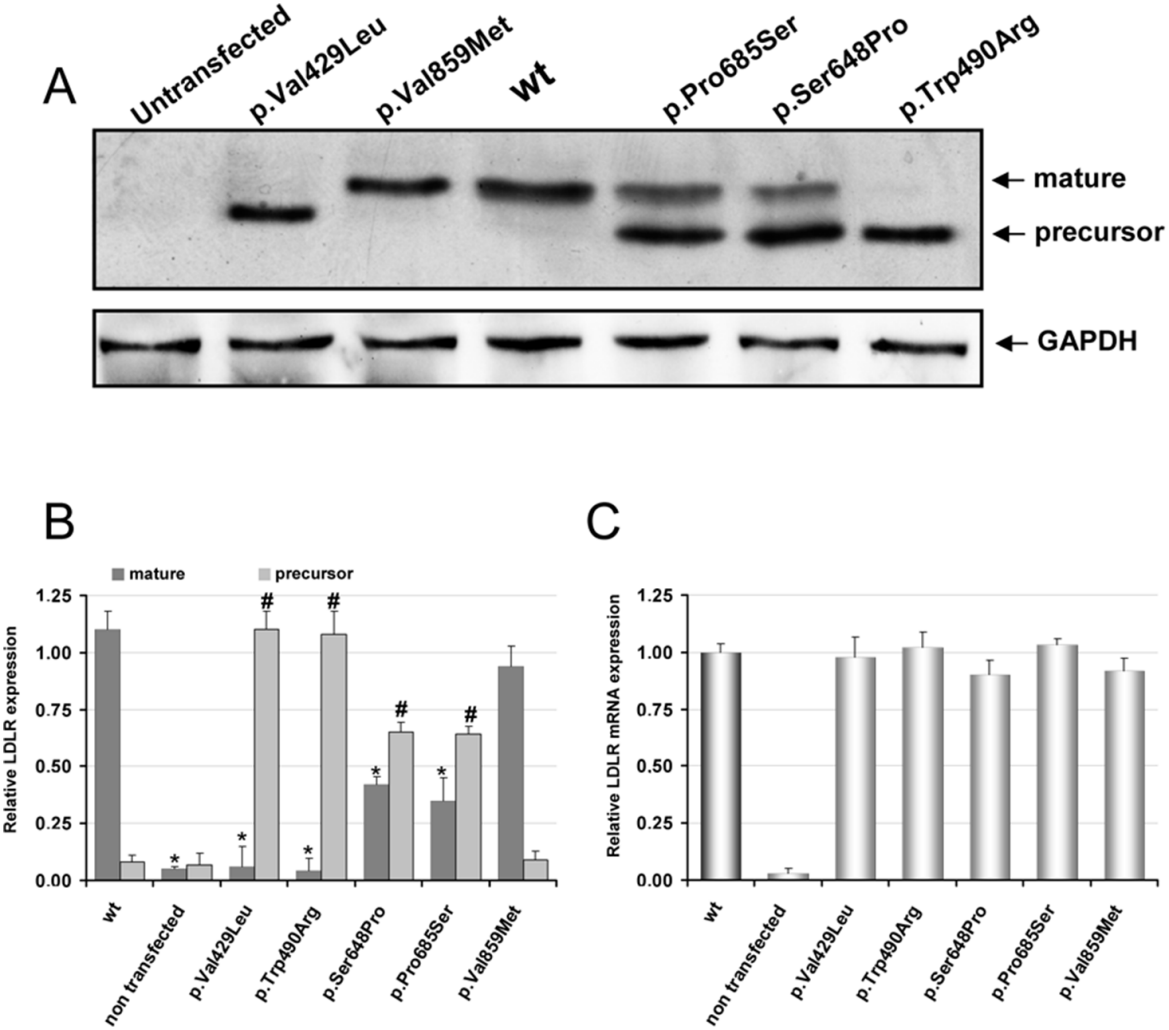
Protein and mRNA expression of wt LDLR and LDLR variants in CHO-*ldlA7* transfected cells. Cells were transfected with the corresponding plasmids carrying the mutations of interest, LDLR was overexpressed for 48 h and then (A) protein expression was analyzed by Western blot, (B) the relative band intensities of both mature and precursor LDLR forms were calculated as the ratio of 160 kDa or 130 kDa LDLR band intensity to that of GAPDH (C) relative LDLR mRNA expression determined by qRT-PCR (normalised to GAPDH). A representative experiment from three independently performed assays is shown in (A). The values in (B) and (C) represent the mean of triplicate determinations (n = 3) and (n = 2), respectively; error bars represent ±SD. Levels of significance were determined by a two-tailed Student’s t-test, and a confidence level of greater than 95% (p<0.05) was used to establish statistical significance. *p<0.001 compared to the LDLR wt 160 kDa band and #p<0.001 compared to LDLR wt 130 kDa band using a Student’s t-test.

Expression of LDLR in the CHO-*ldl*A7 transfected cells was assayed by immunoblotting and qRT-PCR. As shown in Figure 1A (upper panel), only the band corresponding to the LDLR mature form is detected in wt LDLR and p.Val859Met variants (Fig. 1, lanes 4 and 3, respectively); For variants p.Val429Leu and p.Trp490Arg only the band corresponding to the precursor form of the protein was detected (Fig. 1, lanes 2 and 7). In variants p.Ser648Pro and p.Pro685Ser both mature and precursor forms of LDLR were detected, being the expression of the mature protein lower compared to the wt receptor (Fig. 1, lanes 5 and 6). The extent of protein expression was determined by quantitative densitometric analysis using cytosolic GAPDH protein expression as internal control (Figure 1B). LDLR mRNA levels in the transfected cells with wt protein and LDLR variants were determined by qRT-PCR after RNA extraction as described in *Materials and Methods*. As shown in Figure 1C, the relative LDLR mRNA expression (normalised to GAPDH) of the LDLR variants resulted similar than the wt.

### Uptake and degradation of ^125^I-LDL and uptake of FITC-LDL in wt LDLR CHO-*ldlA7* transfected cells

**Figure 2:**
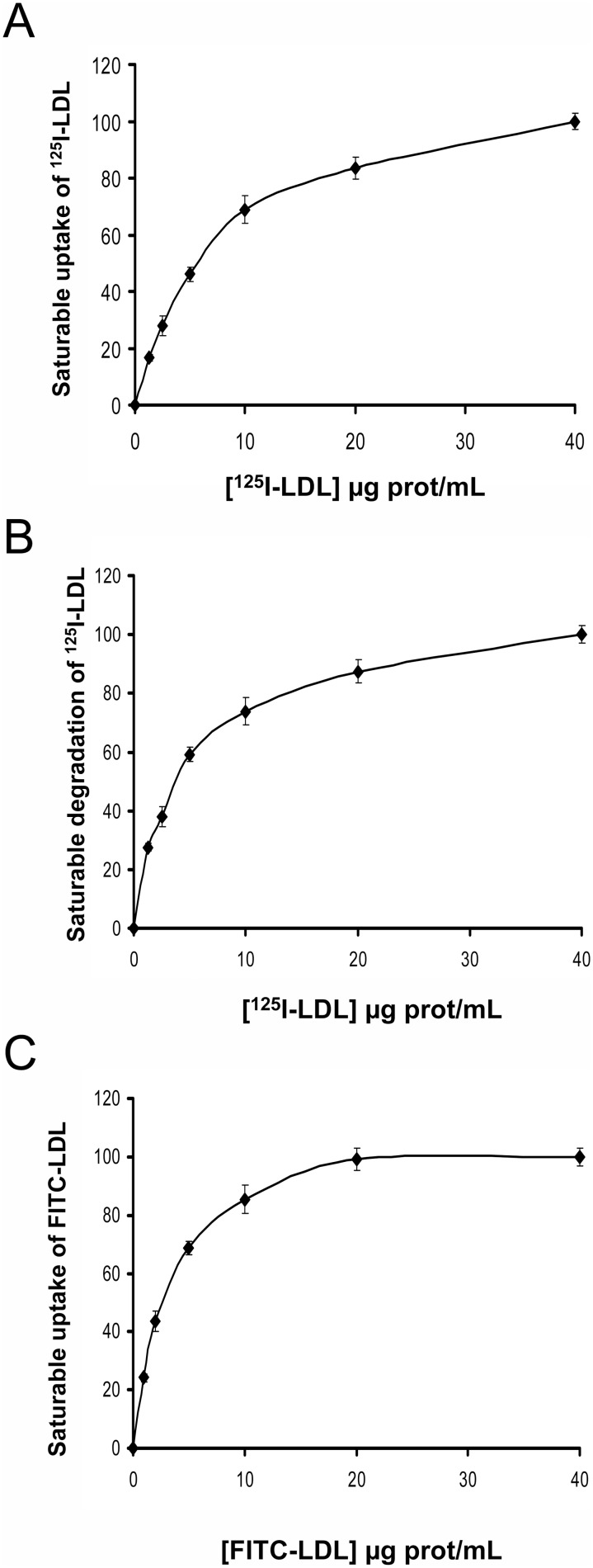
Saturable uptake and degradation of labeled-LDL in CHO-*ldlA7* transfected cells with wt LDLR. (A) saturable uptake of ^125^I-LDL, (B) saturable degradation of ^125^I-LDL and, (C) saturable uptake of FITC-LDL. For analysis of ^125^I-LDL uptake (binding plus internalization) and degradation, CHO-*ldlA7* transfected cells expressing wt LDLR were incubated for 4 h at 37°C with increasing concentrations of ^125^I-LDL as indicated. Subsequently, cells were assayed for cell associated radioactivity (uptake) and degradation of the protein component of LDL. Values were corrected for non-saturable association or degradation determined in the presence of an excess of unlabeled LDL (1 mg/mL). For analysis of FITC-LDL uptake, CHO-*ldlA7* transfected cells expressing wt LDLR were incubated for 4 h at 37°C with increasing concentrations of FITC-LDL as indicated. 10,000 cells were acquired in a Facscalibur and values of LDL uptake were calculated as described in Material and Methods. The values represent the mean of triplicate determinations (n = 3); error bars represent ±SD.

Cell lines expressing wt LDL receptor were assayed for their ability to mediate specific, saturable uptake and degradation of ^125^I-LDL or FITC-LDL uptake. Cells were incubated with labeled-LDL at different concentrations as indicated in *Materials and Methods.* As shown in Fig. 2, saturable activity of LDLR was reached at LDL concentrations above 10 µg/mL LDL for both ^125^I-LDL and FITC-LDL. Statistically significant values of uptake and degradation of ^125^I-LDL (Fig. 2A and B) or uptake of FITC-LDL (Fig. 2C) were determined at concentrations as low as 1 µg/mL. Accordingly, from these results it may be inferred that LDLR uptake activity can be determined by both methods with similar threshold of sensitivity.

### Uptake and degradation of ^125^I-LDL or FITC-LDL uptake by LDLR variants in transfected CHO-*ldlA7* cells

**Figure 3:**
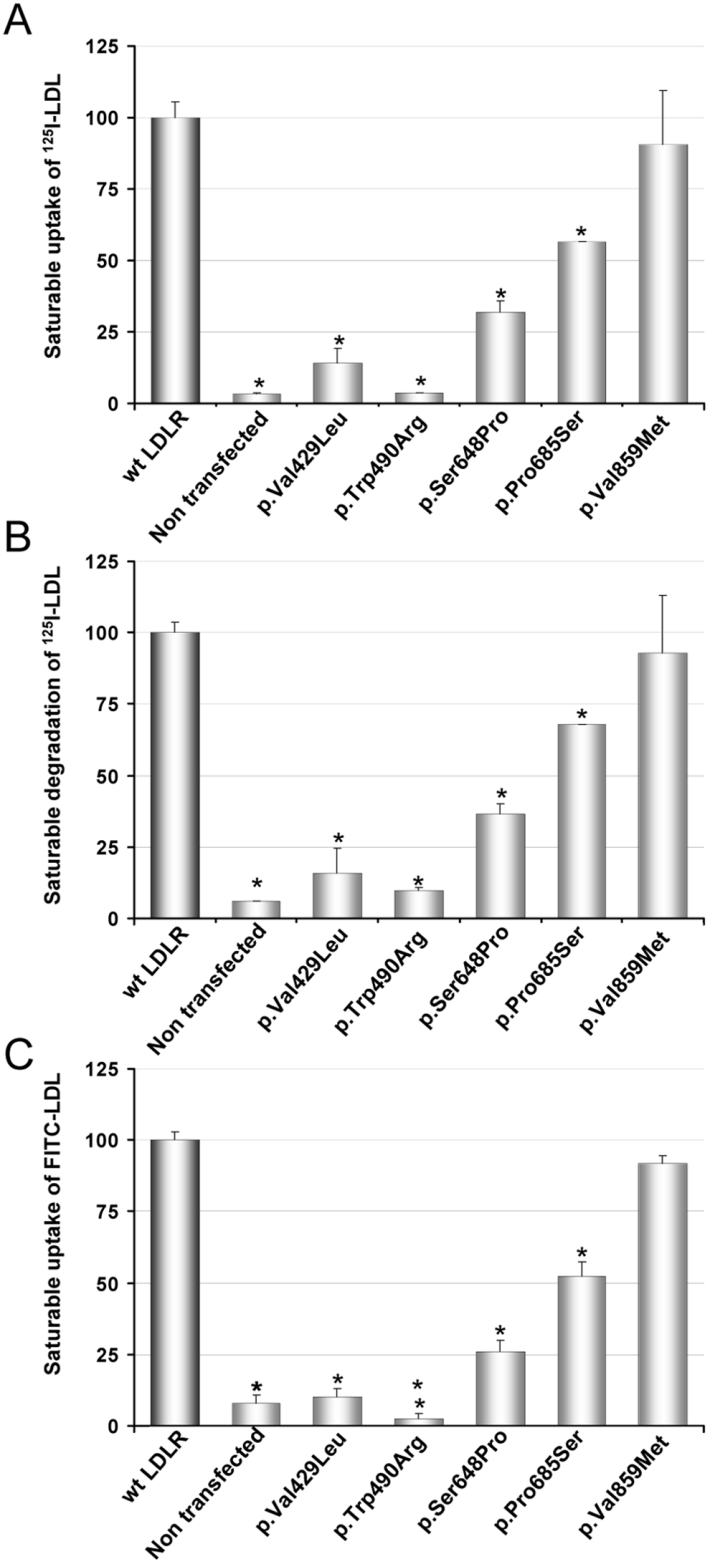
Uptake and degradation of labeled-LDL in CHO-*ldlA7* transfected cells with wt or different LDLR variants. (A) Uptake of ^125^I-LDL, (B) degradation of ^125^I-LDL and, (C) uptake of FITC-LDL. For analysis of ^125^I-LDL uptake (binding plus internalization) and degradation, CHO-*ldlA7* transfected cells expressing wt LDLR were incubated for 4 h at 37°C with 20 µg/mL of ^125^I-LDL as indicated. Subsequently, cells were assayed for cell associated radioactivity (uptake) and degradation of the protein component of LDL. Values were corrected for non-saturable association or degradation determined in the presence of an excess of unlabeled LDL (1 mg/mL). For analysis of FITC-LDL uptake, CHO-*ldlA7* transfected cells expressing wt LDLR were incubated for 4 h at 37°C with 20 µg/mL of FITC-LDL as indicated. 10,000 cells were acquired in a Facscalibur and values of LDL uptake were calculated as described in Material and Methods. The values represent the mean of triplicate determinations (n = 3); error bars represent ±SD. *p<0.001 compared to LDLR wt using a Student’s t-test.

Cell lines expressing wt LDL receptor or the LDLR to p.Val429Leu, p.Trp490Arg, p.Ser648Pro, p.Pro685Ser and p.Val859Met variants were assayed separately by each laboratory for their LDL uptake activities [11]. For this purpose, transfected cells were incubated with 20 µg/mL of the corresponding labeled-LDL for 4 h at 37°C as indicated in *Materials and Methods.* Cells expressing variants p.Val429Leu and p.Trp490Arg showed virtually no LDL uptake or degradation of ^125^I-LDL (Fig. 3A and B) and did not show any statistically significant uptake of FITC-LDL when compared with non transfected CHO-*ldlA7* cells (Fig. 3C). Cells expressing variants p.Ser648Pro and p.Pro685Ser were impaired in both uptake and degradation, showing only 32% and 56%, of ^125^I-LDL uptake capacity, respectively, and 26% and 52% uptake of FITC-LDL, respectively, when compared to cells expressing wt LDLR (Fig. 3A, and C). p.Val859Met variant presented a similar activity than the wt, both determined by uptake of ^125^I-LDL and FITC-LDL (Fig. 3A and C). Once again, we observed similar results when comparing data obtained by radioactivity labeling or by fluorescent labeling.

### Binding of FITC-LDL to LDLR variants in CHO-*ldlA7* cells

**Figure 4:**
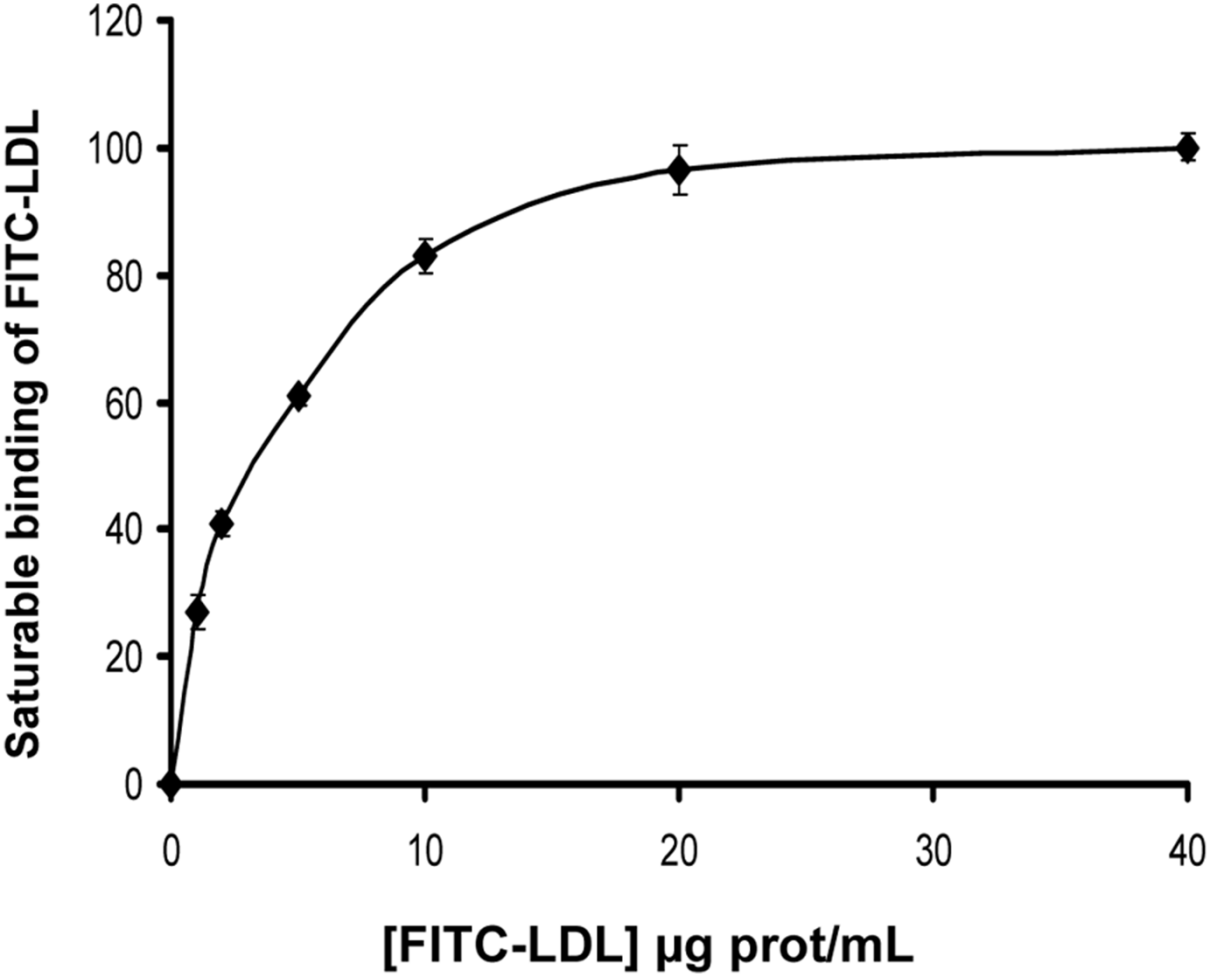
Saturable binding of FITC-LDL in in CHO-*ldlA7* transfected cells with wt LDLR. For analysis of FITC-LDL binding, CHO-*ldlA7* transfected cells expressing wt LDLR were incubated for 4 h at 4°C with increasing concentrations of FITC-LDL as indicated. 10,000 cells were acquired in a Facscalibur and values of LDL uptake were calculated as described in Material and Methods. The values represent the mean of triplicate determinations (n = 3); error bars represent ±SD.

**Figure 5:**
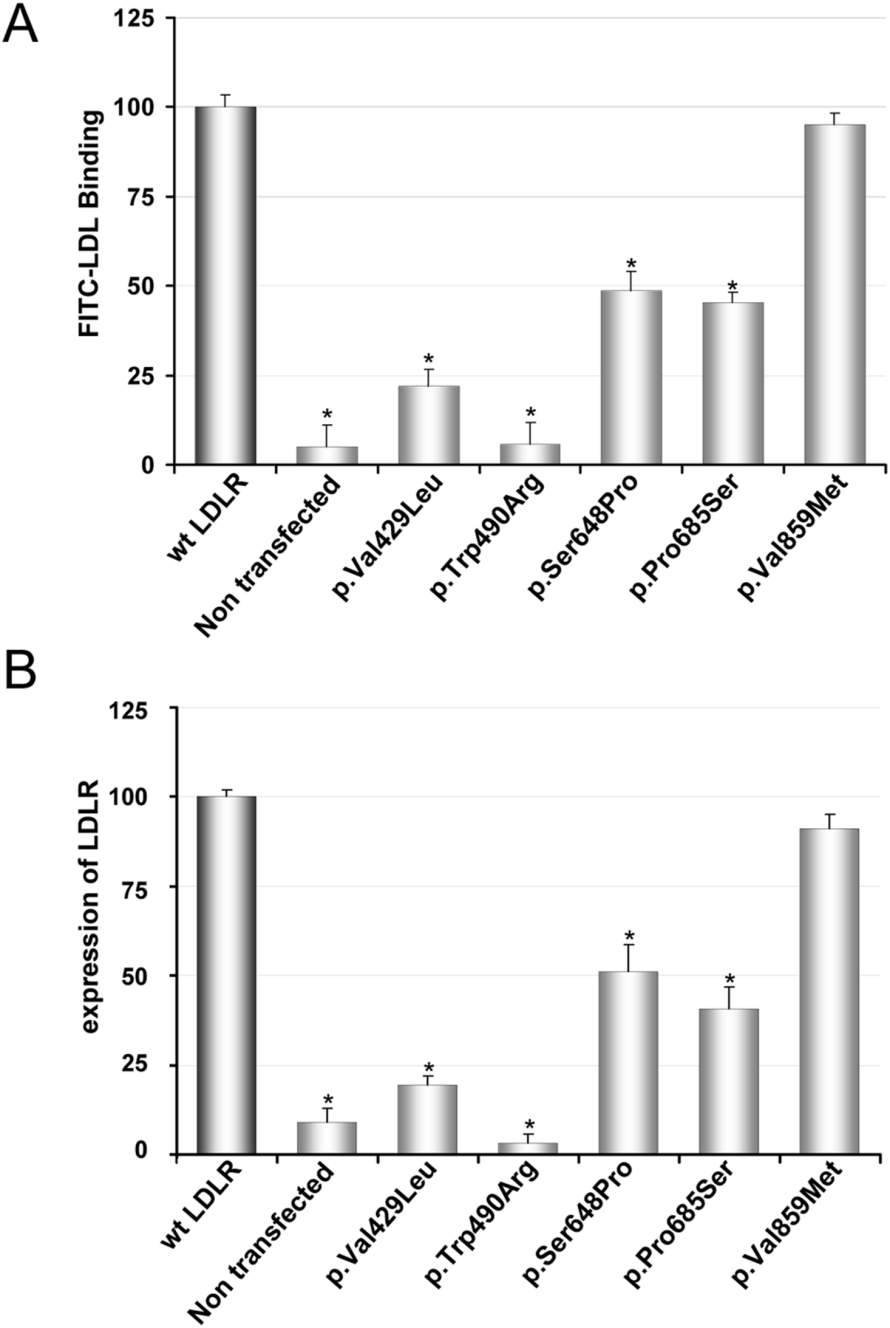
FITC-LDL binding and LDLR expression in CHO-*ldlA7* transfected cells with wt or different LDLR variants. (A) For analysis of FITC-LDL binding, CHO-*ldlA7* transfected cells with different LDLR variants were incubated for 4 h at 4°C with 20 µg/mL of FITC-LDL as indicated. (B) For analysis of LDLR expression, transfected cells were incubated for 48 h and then the LDLR expression at cellular membrane was determined as described in Material and Methods. 10,000 cells were acquired in a Facscalibur and values of LDL uptake were calculated as described in Material and Methods. The values represent the mean of triplicate determinations (n = 3); error bars represent ±SD. *p<0.001 compared to LDLR wt using a Student’s t-test.

In order to explain whether the LDL uptake differences obtained with the LDLR variants were correlated with differences in LDL binding, we used flow cytometry to determine FITC-LDL binding efficiency for each variant. Cells were incubated with 20 µg/mL FITC-LDL for 4 h at 4°C as described in *Materials and Methods*. This LDL concentration was chosen because binding saturation of FITC-LDL is achieved at [LDL] >10 µg/mL (Fig. 4). As depicted in Fig. 5A the variants p.Val429Leu, p.Trp490Arg, p.Ser648Pro and p.Pro685Ser showed a diminished binding capacity respective to wt (wt: 100±5; p.Val429Leu: 22±4, p.Trp490Arg: 5.8±5, p.Ser648Pro: 48±5 and p.Pro685Ser: 45±3) (Fig. 5A), while the binding efficiency of p.Val859Met was similar to the wt (wt: 100±5; p.Val859Met: 95±3, p>0.26). Therefore, the binding data were in agreement with the uptake values obtained for the different LDLR variants.

### Expression of LDLR variants in CHO-*ldlA7* cells

The surface expression of the LDLR was quantified by flow cytometry (Fig. 5B). For p.Val859Met variant the amount of LDLR detected was very similar to the wt LDLR (wt: 100±2; p.Val859Met: 91±4; p>0.15) (Fig. 5B). For p.Pro685Ser and p.Ser648Pro variants LDLR expression was approximately the half with respect to wt (wt: 100±2; p.Pro685Ser: 41±6 and p.Ser648Pro: 51±7). In the case of p.Val429Leu and p.Trp490Arg variants there was not significant receptor expression, which agrees with the data of LDL uptake and binding (Fig. 3 and Fig. 5A).

### Intracellular localization of LDLR variants and class mutation assignment

**Figure 6:**
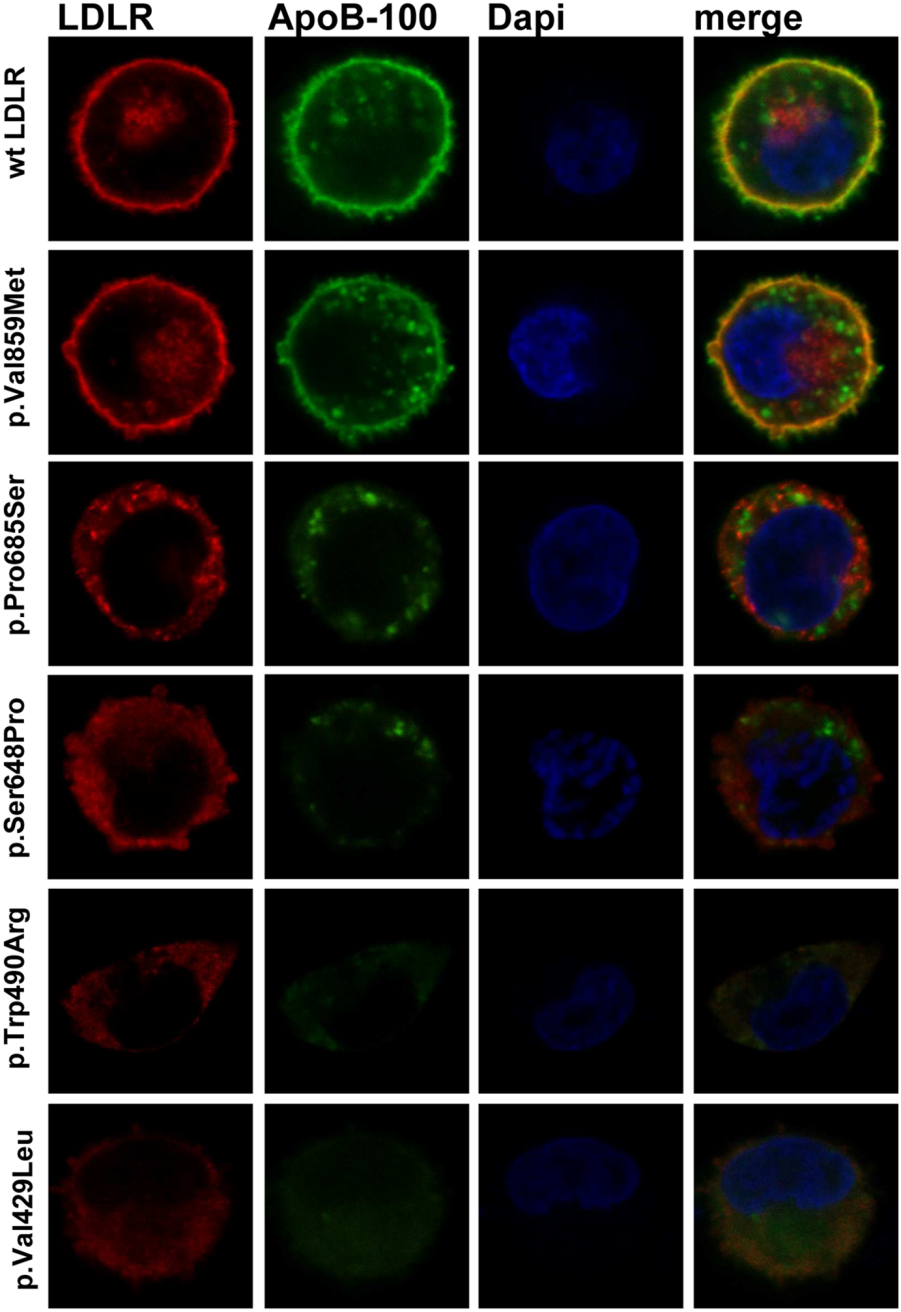
LDLR expression of the different variants at cell surface in CHO-*ldlA7* transfected cells. For confocal analysis of LDLR at cellular membrane and LDL uptake, CHO-*ldlA7* transfected cells were immunostained as described in Material and Methods with anti-hLDLR antibody and anti-ApoB100 antibody. Texas Red and Alexa Fluor 488 labeled secondary antibodies were used to visualize LDLR or LDL, respectively. Dapi was used to stain nuclei. The images show a representative individual cell.

**Figure 7:**
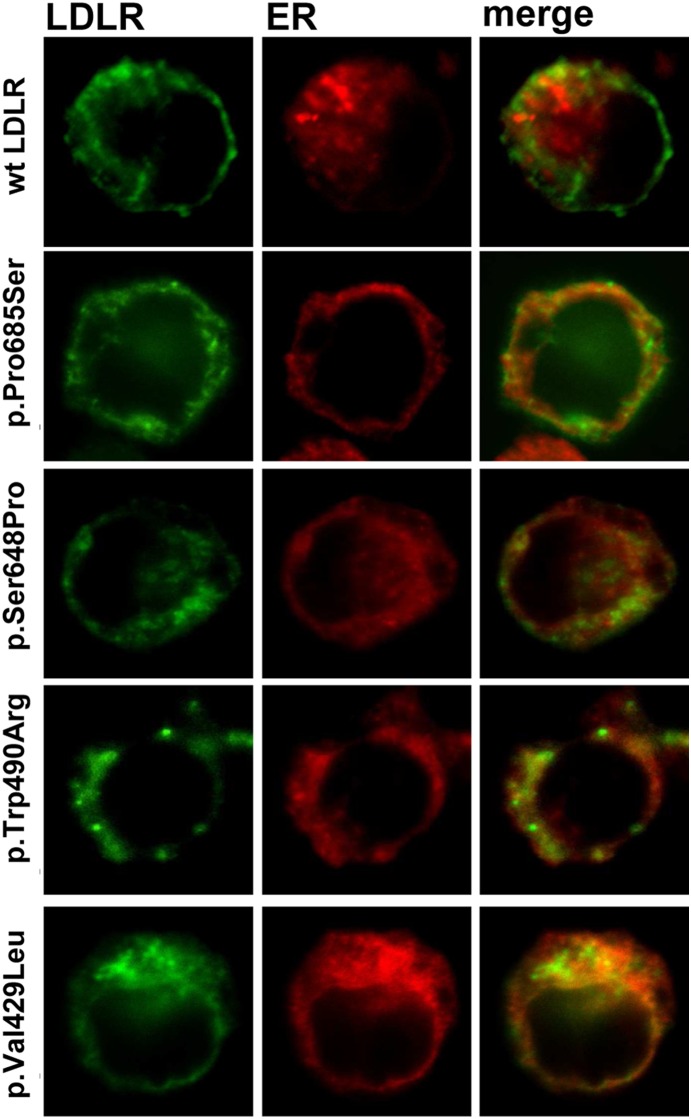
LDLR colocalization wit ER of the different variants in CHO-*ldlA7* transfected cells. For confocal analysis of colocalization of LDLR with ER, CHO-*ldlA7* transfected cells were immunostained as described in Material and Methods with anti-hLDLR antibody and anti-calreticulin antibody. Alexa Fluor 488 and Texas Red labeled secondary antibodies were used to visualize LDLR or calreticulin, respectively. The images show a representative individual cell.

Confocal microscopy was used to corroborate the failure of receptor expression or LDLR class mutation. Cells expressing wt or LDLR variants were incubated with LDL for 4 h and then immunostained with the appropriate antibodies to determine LDLR and LDL localization within the cell. Texas Red or FITC-conjugated secondary antibodies were used to visualize LDLR and LDL, respectively. As shown in Fig. 6, p.Ser648Pro and p.Pro685Ser variants showed a diminished receptor expression and, p.Trp490Arg and p.Val429Leu variants did not express significant amount of receptor. In agreement with the LDLR expression results obtained by cytometry, confocal images of p.Val859Met variant showed a pattern of LDLR expression similar to the wt. Additionally, we analyzed the intracellular localization of these variants to figure out their class defect, thus colocalization of these variants with calreticulin, a specific marker of the endoplasmic reticulum (ER) was assayed (Fig. 7) According to the results, colocalization of p.Trp490Arg and p.Val429Leu with ER indicates that these LDLR variants are class 2a mutant receptors with a completely defective transport from the ER to the Golgi apparatus. Regarding p.Pro685Ser and p.Ser648Pro variants, confocal images showed a partial retention of LDLR in the ER, thus the transport to the membrane is partially blocked indicating that these variants belong to class 2b mutant receptors.

A detailed analysis of p.Trp490Arg (Figure S2) shows that Trp490 residing in the YWTD repeat maintains apolar interactions with the surrounding amino acids which can not be maintained when substituted by Arginine in p.Trp490Arg mutant. Regarding p.Val429Leu, as shown in Figure S3, it is possible that the replacement of Val by a bigger amino acid as leucine does not allow the correct folding of two β-sheets. These effects may be responsible of retention of the two mutants in the ER, thus causing class 2a defects. The partial retention of LDLR in the ER occurring in class 2b mutants p.Ser648Pro and p.Pro685Ser may be related to structural modifications introduced by the amino acid replacement (Figures S4 and S5). Regarding p.Ser648Pro mutant, in wt LDLR, S648 is located in the first β-sheet of the sixth YWTD blade, adjacent to a proline that facilitates a turn to form the central barrel of the β-propeller. The replacement of Ser648 by Pro, leading two consecutive prolines, could disturb in someway the structure of the β-propeller (Figure S4). Pro685, located in the EGF-C domain of the LDL-receptor, has been identified as one of the necessary residues involved in the packing of this domain [31]. It has been described that Pro685 establishes hydrophobic interactions into a groove of the β-propeller with Val529 and Tyr532. As shown in Figure S5, the replacement of Pro685 by a serine could interfere in these interactions due to its polarity.

## Discussion

CHD is the most common type of heart disease and the leading cause of global morbidity and mortality in adults contributing to at least one in every three deaths each year [32]–[34]. FH patients, if untreated, have an increased risk of developing premature CHD but this can be prevented by early identification and consequent implementation of lipid lowering medication. There are a number of criteria for diagnosing FH phenotypically in adults, but clinical identification is not sufficient to identify a FH patient [35]. Increasing emphasis on genetic testing has become a clear example of how new genome technologies can contribute to the benefit of patients, but only the identification of a functional FH-causing mutation in an individual provides a definitive diagnosis. To date, more than 1300 genetic variants in the *LDLR* have been described so far, and approximately 80–90% have been designated as potentially pathogenic [3], [10], [36] although the great majority do not have functional studies [3]. The early and definitive identification of FH is important to improve patient prognosis by the implementation of adequate treatment to prevent premature CHD, especially effective in younger individuals. [37] and, would also save large amounts of money to health services [38].

Nowadays, studies of LDLR functionality *ex vivo* are carried out mainly by radioactivity assays based on measurement of binding, uptake and degradation of ^125^I-labeled LDL or by fluorescence-based procedures. For many years the reference method to estimate LDLR activity has been the radioactive assay [39]. However approaches using fluorescently-labelled LDL have also been described and allow determination of LDL binding and uptake by flow cytometry [11], [14], [30]. Radioactive assay has the advantage of being very sensitive, but it also has serious drawbacks such as the risk of exposure to radioisotopes for experimenters and colleagues, or the difficulties and ethical considerations of nuclear waste elimination procedures. In contrast, the use of fluorimetric assays based on covalent labelling of LDL with fluorophores such as FITC overcomes many of the aforementioned problems associated with radiolabeling. Labeling of LDL with FITC is an inexpensive procedure, approximately 40 times cheaper than ^125^I-labeling, and stable fluorophore-protein complex can be obtained, quickly and simply and radioactivity-handling drawbacks are avoided.

In this work we have validated the fluorimetric assay by comparing LDLR activities of previously validated variants by the reference method [39] with the activities of the same variants determined by FACS. In LDLR activity characterization (binding and uptake) by ^125^I-labeling and FITC-LDL, the results were very similar, both in sensitivity as in accuracy (Fig. 2 and 4). In addition, flow cytometry allowed the quantification of LDLR expression at the cell surface (Fig. 4 and 5), providing a better characterization of the defect associated to each mutation. In fact, by complementation of flow cytometry assays with confocal microscopy, which allows detection of the subcellular localization of the LDLR synthesized, it is possible to assign the class type of each variant studied.

To conclude, the data presented here show that flow cytometry using FITC-labeled LDL is a reliable and accurate methodology to determine LDLR activity. This method combined with the use of confocal microscopy offers the possibility of class type assignment for LDLR mutants, thus contributing to improve the diagnosis of FH. In sum, fluorescence-based methodology is a highly recommendable option to be used for the functional analysis of LDLR variants before reporting any variant for genetic testing.

Furthermore, the increasing genotyping results obtained by the next generation sequencing technique is providing a higher number of variants that will need functional validation. Thus, the validated methodology will be of great importance to validate functionally the described and novel alterations in order to provide an accurate diagnosis of FH.

## Supporting Information

Figure S1 **Specificity of LDLR antibody in in wt LDLR transfected and non transfected CHO-** ***ldl*** **A7 cells.** Cells grown during 48 h were incubated with a mouse primary antibody anti-LDLR or with mouse IgG2b, kappa monoclonal [MPC-11]-isotype control as described in *Materials and Methods*. 10,000 cells were acquired in a Facscalibur and values of LDL uptake were calculated as described in Material and Methods. The values represent the mean of triplicate determinations (n = 3); error bars represent ±SD. *p<0.001 compared to LDLR wt using a Student’s t-test.
(TIF)

Figure S2 **Structure of the blades maintained by the Trp located in the YWTD repeats of the β-propeller.** (A) Hydrophobic contacts of the Trp with the surrounding residues, and the water mediated hydrogen bound between the indol group of tryptophan and one carboxylate group of an adjacent Asp maintain the blade-structures. (B) Replacement of Trp490 by an Asn impairs hydrophobic interactions. This figure was prepared with PyMOL (DeLano scientifics) (PDB:1N7D).
(TIF)

Figure S3 **Structure of the Val429 and surrounding amino acids (A).** Val429 allows the correct packing of the structure maintaining hydrophobic contacts. Leu429 introduces very little change in size, but maybe sufficient to impair a correct folding of the protein (B). This figure was prepared with PyMOL (DeLano scientifics) (PDB:1IJQ).
(TIF)

Figure S4 **Structure of the β-propeller central barrel (A).** The prolines located at the beginning of each β-sheet allow a better turn to form the barrel. Two consecutive prolines could disturb the structure (B). This figure was prepared with PyMOL (DeLano scientifics) (PDB:1N7D) (PDB:3S06).
(TIF)

Figure S5 **Structure of the interface of β-propeller in green and EGF-C in blue (A).** Pro685 establishes hydrophobic contacts with β-propeller. Ser685, due to its polarity could disturb this contact (B). This figure was prepared with PyMOL (DeLano scientifics) (PDB:1N7D) (PDB:3S06).
(TIF)

## References

[1] Goldstein JL HH, Brown MS (2001) Familial hypercholesterolemia; C.R. Scriver ALB, W.S. Sly and D. Valle, Editors, editor. New York McGraw-Hill.

[2] Ned RM, Sijbrands EJ (2011) Cascade Screening for Familial Hypercholesterolemia (FH). PLoS Curr 3: RRN1238. 21633520 10.1371/currents.RRN1238 PMC3102597

[3] Usifo E, Leigh SE, Whittall RA, Lench N, Taylor A, et al (2012) Low-density lipoprotein receptor gene familial hypercholesterolemia variant database: update and pathological assessment. Ann Hum Genet 76: 387–401. 22881376 10.1111/j.1469-1809.2012.00724.x

[4] Hobbs HH, Leitersdorf E, Goldstein JL, Brown MS, Russell DW (1988) Multiple crm- mutations in familial hypercholesterolemia. Evidence for 13 alleles, including four deletions. J Clin Invest 81: 909–917. 3343347 10.1172/JCI113402 PMC442544

[5] Goldstein JL, Brown MS, Anderson RG, Russell DW, Schneider WJ (1985) Receptor-mediated endocytosis: concepts emerging from the LDL receptor system. Annu Rev Cell Biol 1: 1–39. 2881559 10.1146/annurev.cb.01.110185.000245

[6] Hobbs HH, Brown MS, Goldstein JL (1992) Molecular genetics of the LDL receptor gene in familial hypercholesterolemia. Hum Mutat 1: 445–466. 1301956 10.1002/humu.1380010602

[7] Davis CG, Lehrman MA, Russell DW, Anderson RG, Brown MS, et al (1986) The J.D. mutation in familial hypercholesterolemia: amino acid substitution in cytoplasmic domain impedes internalization of LDL receptors. Cell 45: 15–24. 3955657 10.1016/0092-8674(86)90533-7

[8] Lehrman MA, Goldstein JL, Brown MS, Russell DW, Schneider WJ (1985) Internalization-defective LDL receptors produced by genes with nonsense and frameshift mutations that truncate the cytoplasmic domain. Cell 41: 735–743. 3924410 10.1016/s0092-8674(85)80054-4

[9] Beglova N, Jeon H, Fisher C, Blacklow SC (2004) Cooperation between fixed and low pH-inducible interfaces controls lipoprotein release by the LDL receptor. Mol Cell 16: 281–292. 15494314 10.1016/j.molcel.2004.09.038

[10] Leigh SE, Foster AH, Whittall RA, Hubbart CS, Humphries SE (2008) Update and analysis of the University College London low density lipoprotein receptor familial hypercholesterolemia database. Ann Hum Genet 72: 485–498. 18325082 10.1111/j.1469-1809.2008.00436.x

[11] Silva S, Alves AC, Patel D, Malho R, Soutar AK, et al (2012) In vitro functional characterization of missense mutations in the LDLR gene. Atherosclerosis 225: 128–134. 23021490 10.1016/j.atherosclerosis.2012.08.017

[12] Knight BL, Soutar AK (1982) Changes in the metabolism of modified and unmodified low-density lipoproteins during the maturation of cultured blood monocyte-macrophages from normal and homozygous familial hypercholesterolaemic subjects. Eur J Biochem 125: 407–413. 7117242 10.1111/j.1432-1033.1982.tb06698.x

[13] Leren TP, Tonstad S, Gundersen KE, Bakken KS, Rodningen OK, et al (1997) Molecular genetics of familial hypercholesterolaemia in Norway. J Intern Med 241: 185–194. 9104431 10.1046/j.1365-2796.1997.78119000.x

[14] Ranheim T, Kulseth MA, Berge KE, Leren TP (2006) Model system for phenotypic characterization of sequence variations in the LDL receptor gene. Clin Chem 52: 1469–1479. 16740646 10.1373/clinchem.2006.068627

[15] Romano M, Di Taranto MD, Mirabelli P, D’Agostino MN, Iannuzzi A, et al (2011) An improved method on stimulated T-lymphocytes to functionally characterize novel and known LDLR mutations. J Lipid Res 52: 2095–2100. 21865347 10.1194/jlr.D017772 PMC3196240

[16] Sun XM, Patel DD, Knight BL, Soutar AK (1997) Comparison of the genetic defect with LDL-receptor activity in cultured cells from patients with a clinical diagnosis of heterozygous familial hypercholesterolemia. The Familial Hypercholesterolaemia Regression Study Group. Arterioscler Thromb Vasc Biol 17: 3092–3101. 9409298 10.1161/01.atv.17.11.3092

[17] Tada H, Kawashiri MA, Noguchi T, Mori M, Tsuchida M, et al (2009) A novel method for determining functional LDL receptor activity in familial hypercholesterolemia: application of the CD3/CD28 assay in lymphocytes. Clin Chim Acta 400: 42–47. 19013141 10.1016/j.cca.2008.10.010

[18] den Dunnen JT, Antonarakis SE (2000) Mutation nomenclature extensions and suggestions to describe complex mutations: a discussion. Hum Mutat 15: 7–12. 10612815 10.1002/(SICI)1098-1004(200001)15:1<7::AID-HUMU4>3.0.CO;2-N

[19] Taschner PE, den Dunnen JT (2011) Describing structural changes by extending HGVS sequence variation nomenclature. Hum Mutat 32: 507–511. 21309030 10.1002/humu.21427

[20] Adzhubei IA, Schmidt S, Peshkin L, Ramensky VE, Gerasimova A, et al (2010) A method and server for predicting damaging missense mutations. Nat Methods 7: 248–249. 20354512 10.1038/nmeth0410-248 PMC2855889

[21] Ng PC, Henikoff S (2003) SIFT: Predicting amino acid changes that affect protein function. Nucleic Acids Res 31: 3812–3814. 12824425 10.1093/nar/gkg509 PMC168916

[22] Gonzalez-Perez A, Lopez-Bigas N (2011) Improving the assessment of the outcome of nonsynonymous SNVs with a consensus deleteriousness score, Condel. Am J Hum Genet 88: 440–449. 21457909 10.1016/j.ajhg.2011.03.004 PMC3071923

[23] Schwarz JM, Rodelsperger C, Schuelke M, Seelow D (2010) MutationTaster evaluates disease-causing potential of sequence alterations. Nat Methods 7: 575–576. 20676075 10.1038/nmeth0810-575

[24] Grantham R (1974) Amino acid difference formula to help explain protein evolution. Science 185: 862–864. 4843792 10.1126/science.185.4154.862

[25] Pollard KS, Hubisz MJ, Rosenbloom KR, Siepel A (2010) Detection of nonneutral substitution rates on mammalian phylogenies. Genome Res 20: 110–121. 19858363 10.1101/gr.097857.109 PMC2798823

[26] Goldstein JL, Brown MS (1974) Binding and degradation of low density lipoproteins by cultured human fibroblasts. Comparison of cells from a normal subject and from a patient with homozygous familial hypercholesterolemia. J Biol Chem 249: 5153–5162. 4368448

[27] Soutar AK, Knight BL, Patel DD (1989) Identification of a point mutation in growth factor repeat C of the low density lipoprotein-receptor gene in a patient with homozygous familial hypercholesterolemia that affects ligand binding and intracellular movement of receptors. Proc Natl Acad Sci U S A 86: 4166–4170. 2726768 10.1073/pnas.86.11.4166 PMC287410

[28] Dardik R, Varon D, Tamarin I, Zivelin A, Salomon O, et al (2000) Homocysteine and oxidized low density lipoprotein enhanced platelet adhesion to endothelial cells under flow conditions: distinct mechanisms of thrombogenic modulation. Thromb Haemost 83: 338–344. 10739396

[29] Hed J, Hallden G, Johansson SG, Larsson P (1987) The use of fluorescence quenching in flow cytofluorometry to measure the attachment and ingestion phases in phagocytosis in peripheral blood without prior cell separation. J Immunol Methods 101: 119–125. 3112235 10.1016/0022-1759(87)90224-9

[30] Etxebarria A, Palacios L, Stef M, Tejedor D, Uribe KB, et al (2012) Functional characterization of splicing and ligand-binding domain variants in the LDL receptor. Hum Mutat 33: 232–243. 21990180 10.1002/humu.21630

[31] Jeon H, Meng W, Takagi J, Eck MJ, Springer TA, et al (2001) Implications for familial hypercholesterolemia from the structure of the LDL receptor YWTD-EGF domain pair. Nat Struct Biol 8: 499–504. 11373616 10.1038/88556

[32] Minino AM, Xu J, Kochanek KD, Tejada-Vera B (2009) Death in the United States, 2007. NCHS Data Brief: 1–8. 20018136

[33] Terzic A, Waldman S (2011) Chronic diseases: the emerging pandemic. Clin Transl Sci 4: 225–226. 21707955 10.1111/j.1752-8062.2011.00295.x PMC5439863

[34] Waldman SA, Terzic A (2011) Cardiovascular health: the global challenge. Clin Pharmacol Ther 90: 483–485. 21934716 10.1038/clpt.2011.213

[35] Alves AC, Medeiros AM, Francisco V, Gaspar IM, Rato Q, et al (2010) Molecular diagnosis of familial hypercholesterolemia: an important tool for cardiovascular risk stratification. Rev Port Cardiol 29: 907–921. 20964105

[36] Haase A, Goldberg AC (2012) Identification of people with heterozygous familial hypercholesterolemia. Curr Opin Lipidol 23: 282–289. 22801386 10.1097/MOL.0b013e3283556c33

[37] Nordestgaard BG, Chapman MJ, Humphries SE, Ginsberg HN, Masana L, et al (2013) Familial hypercholesterolaemia is underdiagnosed and undertreated in the general population: guidance for clinicians to prevent coronary heart disease: consensus statement of the European Atherosclerosis Society. Eur Heart J 34: 3478–3490a. 23956253 10.1093/eurheartj/eht273 PMC3844152

[38] Brice P, Burton H, Edwards CW, Humphries SE, Aitman TJ (2013) Familial hypercholesterolaemia: a pressing issue for European health care. Atherosclerosis 231: 223–226. 24267231 10.1016/j.atherosclerosis.2013.09.019

[39] Brown MS, Goldstein JL (1975) Regulation of the activity of the low density lipoprotein receptor in human fibroblasts. Cell 6: 307–316. 212203 10.1016/0092-8674(75)90182-8

