# Supplementary figures and images for: Advantages and Versatility of Fluorescence-Based Methodology to Characterize the Functionality of LDLR and Class Mutation Assignment
Source: PLoS One. 2014 Nov 11;9(11):e112677. doi: 10.1371/journal.pone.0112677 (PMC4227843; doi:10.1371/journal.pone.0112677)

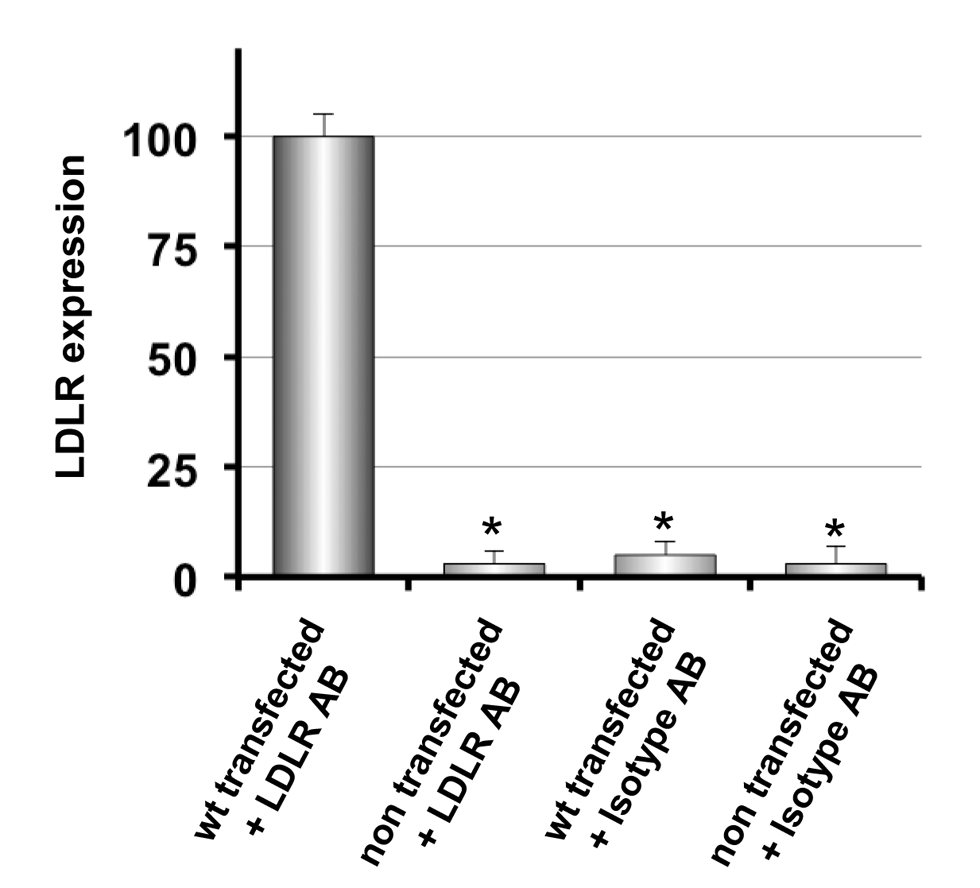

Supplement: Figure S1 — Specificity of LDLR antibody in in wt LDLR transfected and non transfected CHO- ldl A7 cells. Cells grown during 48 h were incubated with a mouse primary antibody anti-LDLR or with mouse IgG2b, kappa monoclonal [MPC-11]-isotype control as described in Materials and Methods . 10,000 cells were acquired in a Facscalibur and values of LDL uptake were calculated as described in Material and Methods. The values represent the mean of triplicate determinations (n = 3); error bars represent ±SD. *p<0.001 compared to LDLR wt using a Student’s t-test. (TIF) [file pone.0112677.s001.tif]

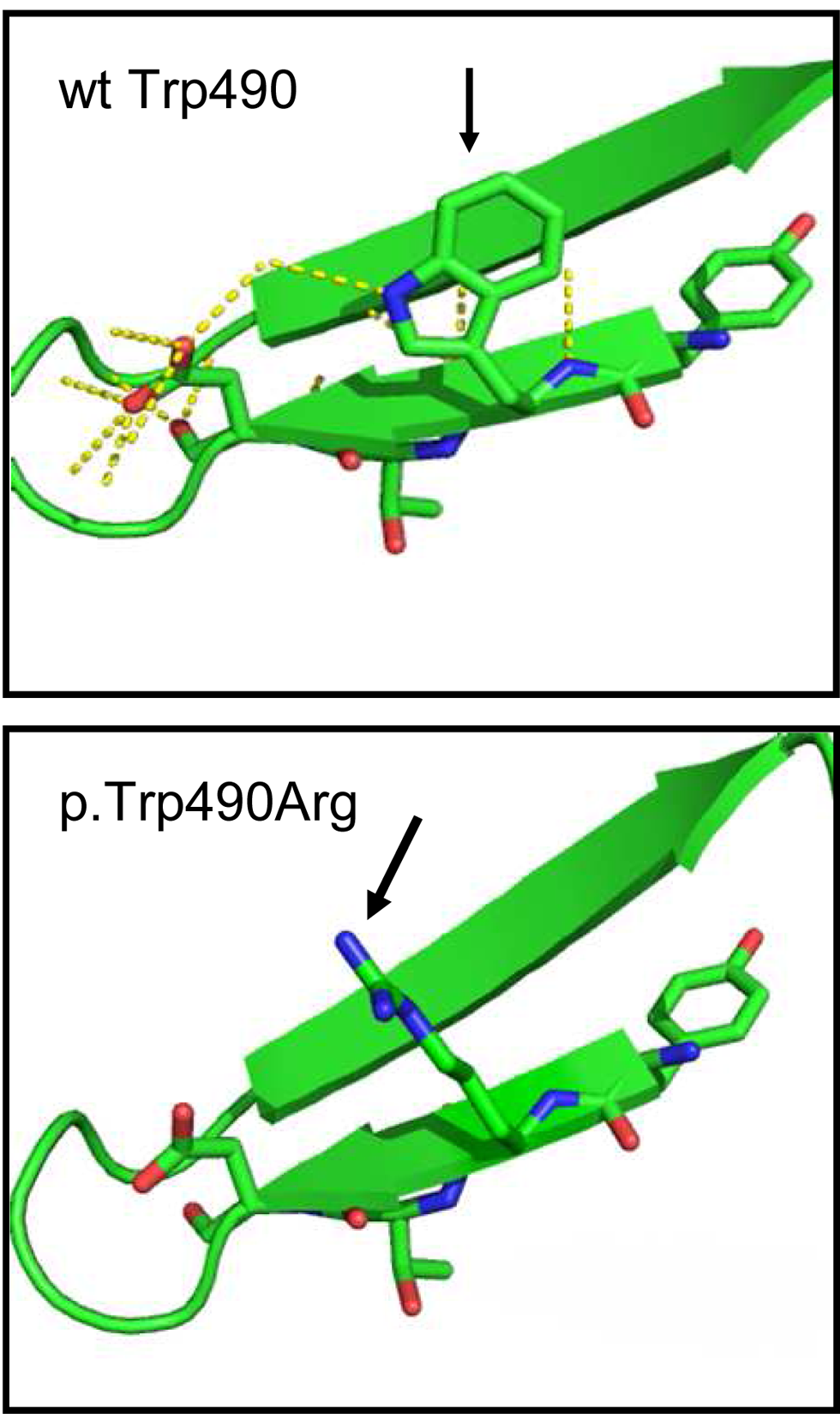

Supplement: Figure S2 — Structure of the blades maintained by the Trp located in the YWTD repeats of the β-propeller. (A) Hydrophobic contacts of the Trp with the surrounding residues, and the water mediated hydrogen bound between the indol group of tryptophan and one carboxylate group of an adjacent Asp maintain the blade-structures. (B) Replacement of Trp490 by an Asn impairs hydrophobic interactions. This figure was prepared with PyMOL (DeLano scientifics) (PDB:1N7D). (TIF) [file pone.0112677.s002.tif]

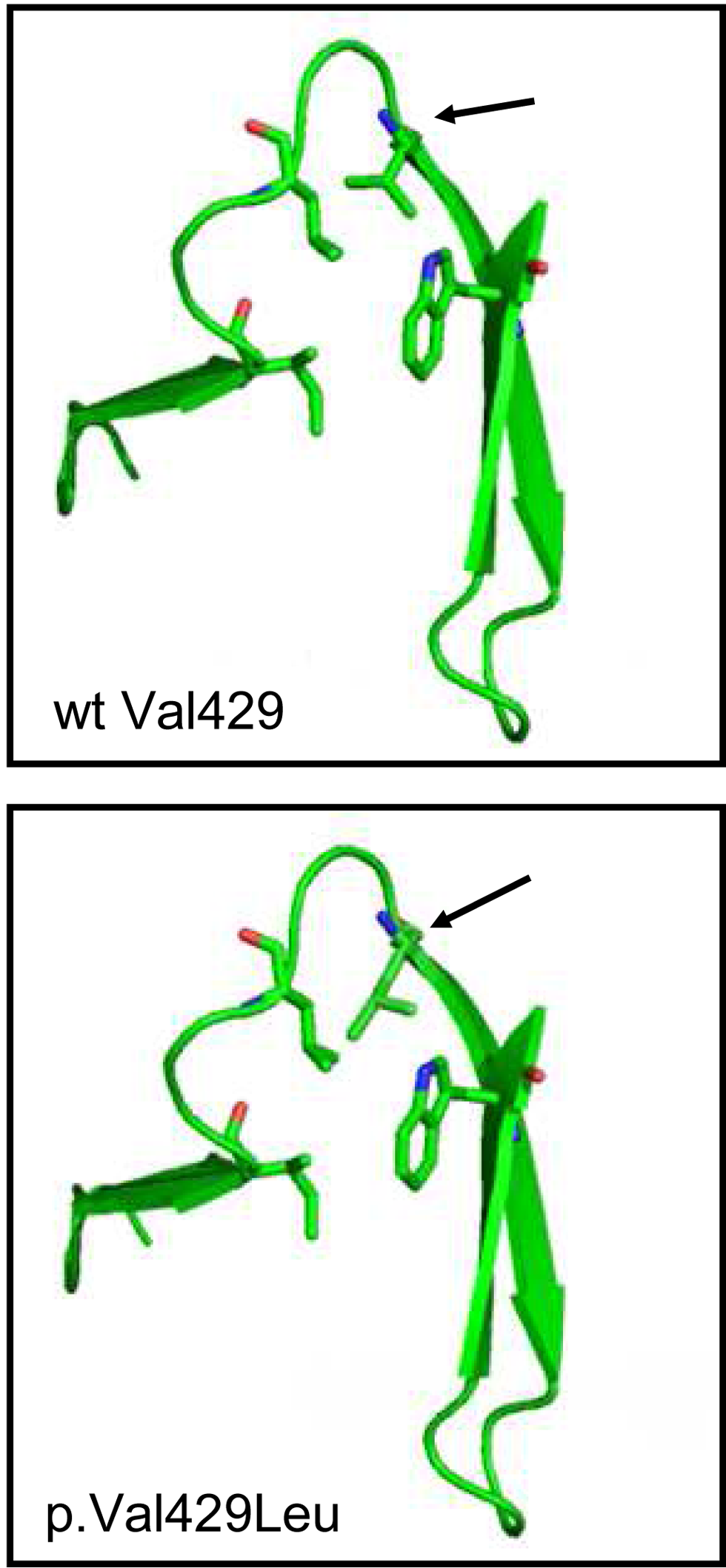

Supplement: Figure S3 — Structure of the Val429 and surrounding amino acids (A). Val429 allows the correct packing of the structure maintaining hydrophobic contacts. Leu429 introduces very little change in size, but maybe sufficient to impair a correct folding of the protein (B). This figure was prepared with PyMOL (DeLano scientifics) (PDB:1IJQ). (TIF) [file pone.0112677.s003.tif]

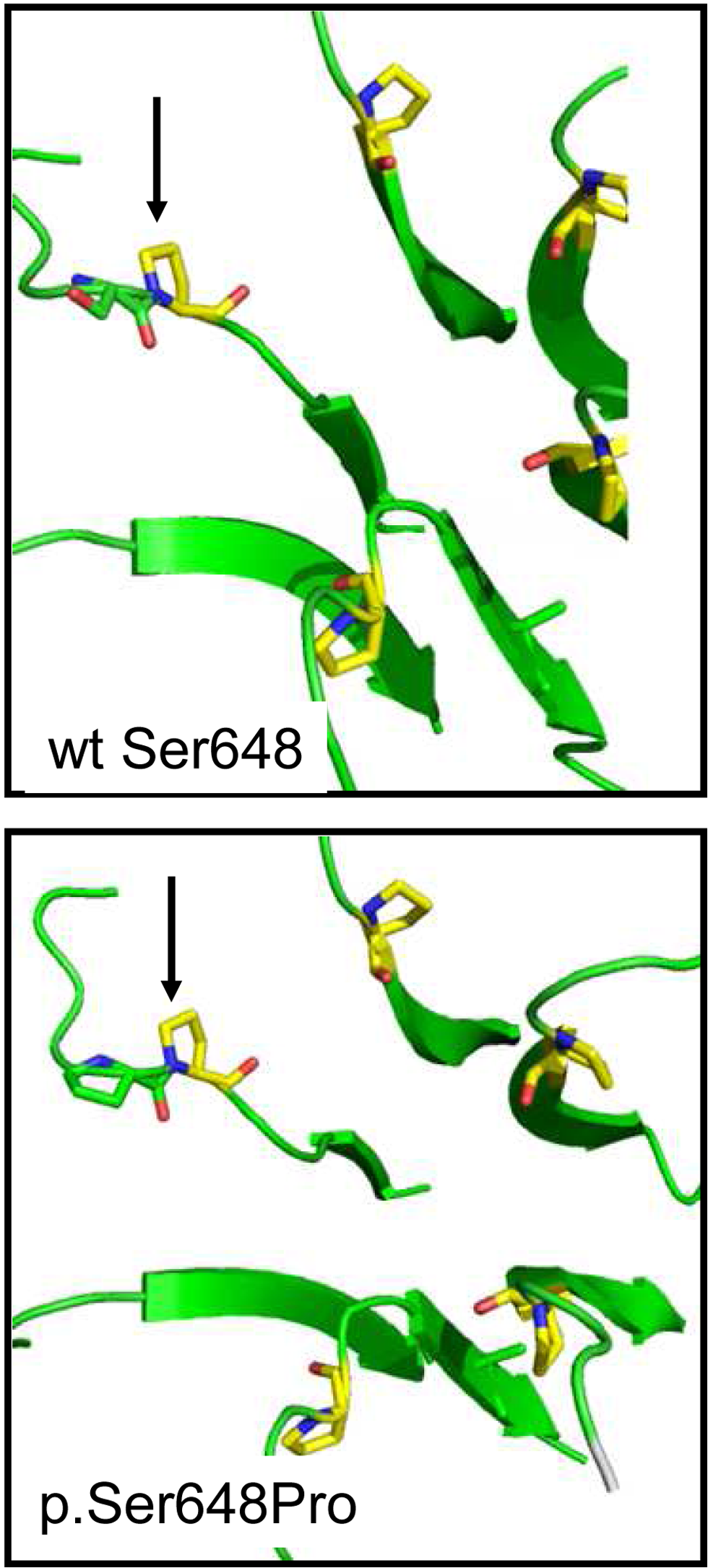

Supplement: Figure S4 — Structure of the β-propeller central barrel (A). The prolines located at the beginning of each β-sheet allow a better turn to form the barrel. Two consecutive prolines could disturb the structure (B). This figure was prepared with PyMOL (DeLano scientifics) (PDB:1N7D) (PDB:3S06). (TIF) [file pone.0112677.s004.tif]

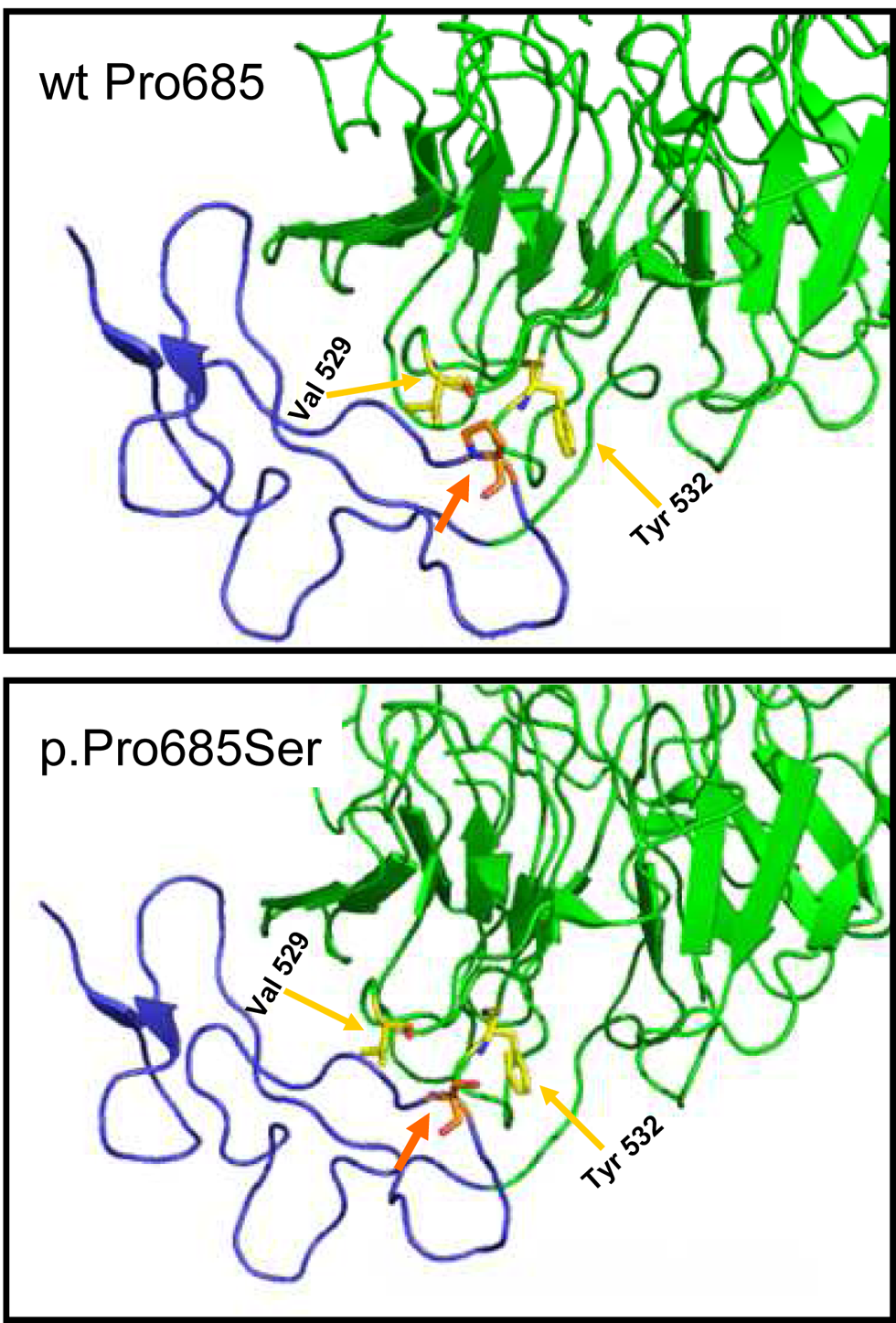

Supplement: Figure S5 — Structure of the interface of β-propeller in green and EGF-C in blue (A). Pro685 establishes hydrophobic contacts with β-propeller. Ser685, due to its polarity could disturb this contact (B). This figure was prepared with PyMOL (DeLano scientifics) (PDB:1N7D) (PDB:3S06). (TIF) [file pone.0112677.s005.tif]
